# A comparative study on the unified model based multifactor dimensionality reduction methods for identifying gene-gene interactions associated with the survival phenotype

**DOI:** 10.1186/s13040-021-00248-9

**Published:** 2021-03-01

**Authors:** Jung Wun Lee, Seungyeoun Lee

**Affiliations:** 1 Department of Statistics, University of Connecticut, Storrs, CT USA; 2 Department of Mathematics and Statistics, Sejong University, 209 Neungdong-ro, Gwangjin-gu, Seoul, 05006 South Korea

**Keywords:** Survival time, Cox model, Multifactor dimensionality reduction method, Gene-gene interaction, Unified model-based method, Kaplan-Meier estimate

## Abstract

**Background:**

For gene-gene interaction analysis, the multifactor dimensionality reduction (MDR) method has been widely employed to reduce multi-levels of gene-gene interactions into high- or low-risk groups using a binary attribute. For the survival phenotype, the Cox-MDR method has been proposed using a martingale residual of a Cox model since Surv-MDR was first proposed using a log-rank test statistic. Recently, the KM-MDR method was proposed using the Kaplan-Meier median survival time as a classifier. All three methods used the cross-validation procedure to identify single nucleotide polymorphism (SNP) using SNP interactions among all possible SNP pairs. Furthermore, these methods require the permutation test to verify the significance of the selected SNP pairs. However, the unified model-based multifactor dimensionality reduction method (UM-MDR) overcomes this shortcoming of MDR by unifying the significance testing with the MDR algorithm within the framework of the regression model. Neither cross-validation nor permutation testing is required to identify SNP by SNP interactions in the UM-MDR method. The UM-MDR method comprises two steps: in the first step, multi-level genotypes are classified into high- or low-risk groups, and an indicator variable for the high-risk group is defined. In the second step, the significance of the indicator variable of the high-risk group is tested in the regression model included with other adjusting covariates. The Cox-UMMDR method was recently proposed by combining Cox-MDR with UM-MDR to identify gene-gene interactions associated with the survival phenotype. In this study, we propose two simple methods either by combining KM-MDR with UM-MDR, called KM-UMMDR or by modifying Cox-UMMDR by adjusting for the covariate effect in step 1, rather than in step 2, a process called Cox2-UMMDR. The KM-UMMDR method allows the covariate effect to be adjusted for in the regression model of step 2, although KM-MDR cannot adjust for the covariate effect in the classification procedure of step 1. In contrast, Cox2-UMMDR differs from Cox-UMMDR in the sense that the martingale residuals are obtained from a Cox model by adjusting for the covariate effect in step 1 of Cox2-UMMDR whereas Cox-UMMDR adjusts for the covariate effect in the regression model in step 2. We performed simulation studies to compare the power of several methods such as KM-UMMDR, Cox-UMMDR, Cox2-UMMDR, Cox-MDR, and KM-MDR by considering the effect of covariates and the marginal effect of SNPs. We also analyzed a real example of Korean leukemia patient data for illustration and a short discussion is provided.

**Results:**

In the simulation study, two different scenarios are considered: the first scenario compares the power of the cases with and without the covariate effect. The second scenario is to compare the power of cases with the main effect of SNPs versus without the main effect of SNPs. From the simulation results, Cox-UMMDR performs the best across all scenarios among KM-UMMDR, Cox2-UMMDR, Cox-MDR and KM-MDR. As expected, both Cox-UMMDR and Cox-MDR perform better than KM-UMMDR and KM-MDR when a covariate effect exists because the former adjusts for the covariate effect but the latter cannot. However, Cox2-UMMDR behaves similarly to KM-UMMDR and KM-MDR even though there is a covariate effect. This implies that the covariate effect would be more efficiently adjusted for in the regression model of the second step rather than under the classification procedure of the first step. When there is a main effect of any SNP, Cox-UMMDR, Cox2-UMMDR and KM-UMMDR perform better than Cox-MDR and KM-MDR if the main effects of SNPs are properly adjusted for in the regression model. From the simulation results of two different scenarios, Cox-UMMDR seems to be the most robust when there is either any covariate effect adjusting for or any SNP that has a main effect on the survival phenotype. In addition, the power of all methods decreased as the censoring fraction increased from 0.1 to 0.3, as heritability increased. The power of all methods seems to be greater under MAF = 0.2 than under MAF = 0.4. For illustration, both KM-UMMDR and Cox2-UMMDR were applied to identify SNP by SNP interactions with the survival phenotype to a real dataset of Korean leukemia patients.

**Conclusion:**

Both KM-UMMDR and Cox2-UMMDR were easily implemented by combining KM-MDR and Cox-MDR with UM-MDR, respectively, to detect significant gene-gene interactions associated with survival time without cross-validation and permutation testing. The simulation results demonstrate the utility of KM-UMMDR, Cox2-UMMDR and Cox-UMMDR, which outperforms Cox-MDR and KM-MDR when some SNPs with only marginal effects might mask the detection of causal epistasis. In addition, Cox-UMMDR, Cox2-UMMDR and Cox-MDR performed better than KM-UMMDR and KM-MDR when there were potentially confounding covariate effects.

## Introduction

With the advent of high-throughput genotyping techniques, a large amount of genotype data has been analyzed in genome-wide association studies. Among these, single nucleotide polymorphisms (SNPs) can modify many phenotypes, including cancer progression, responses to varying levels of drugs and survival outcomes. Numerous statistical methods for genome-wide association studies (GWAS) have been developed to identify susceptibility genes by analyzing these data for single SNP effects. Since the first published GWAS on age-related macular degeneration [1], the GWAS Catalog now contains 179,364 SNP-trait associations with 120,219 SNPs based on 4493 publications since March 2020 (www.ebi.ac.uk/gwas). However, the problem of missing heritability exists because a small proportion of heritability has been explained for the common and complex human diseases [2–4].

For the missing heritability problem, many researchers have focused on the challenge of identifying SNP by SNP interactions because complex diseases are associated with multiple genes and their interactions [3]. Most parametric statistical methods such as logistic regression and ordinary regression models, have difficulty dealing with high-dimensional data because the number of variables exponentially increases with higher-order SNP by SNP interactions. One solution to this problem is to collect a large number of samples which yields a robust estimate of interaction effects. However, this is often far from reality because of the expensive sampling cost. An alternative solution is reducing the high dimensionality of multi-genotypes to a very low level, such as one dimension. The multifactor dimensionality reduction (MDR) method was proposed by Ritchie et al. [5] as a new statistical and computational method to analyze gene-gene interactions in genetic studies. The main principle of MDR is to reduce multi-dimensional genotypes into one-dimensional binary attributes, in which multi-level genotypes of SNPs are classified into either high- or low-risk groups, using a ratio of cases and controls in case-control studies. The MDR algorithm then determines the best pair of SNPs among all possible SNP combinations, yielding the maximum balanced accuracy through a cross-validation procedure. The MDR mechanism can apply higher-order interactions such as two-way, and three-way interactions, because all combinations of multi-way interactions can be reduced to either high or low risk groups using the appropriate classification rules.

The key algorithm of MDR has been extensively applied to quantitative traits and survival phenotypes because it was originally proposed for case-control studies. For a prospective cohort study, the first approach, called Surv-MDR, was proposed by Gui et al. [6] followed by Cox-MDR [7], AFT-MDR [8], and KM-MDR [9]. All these methods follow the same procedure as in the original MDR except for using different classification rules, which are appropriate for the survival phenotype. For example, Surv-MDR uses a log-rank test statistic, whereas Cox-MDR uses a martingale residual of the Cox model, AFT-MDR uses a standardized residual of an accelerated failure time (AFT) model and KM-MDR uses the Kaplan-Meier median survival time. In previous simulation studies [7–9], in which the powers of these methods were compared, both Surv-MDR and KM-MDR performed better than both Cox-MDR and AFT-MDR when there was no covariate effect whereas both Cox-MDR and AFT-MDR had superior power than both Surv-MDR and KM-MDR when any covariate effect existed. This is because both Surv-MDR and KM-MDR are nonparametric and cannot adjust for any confounding covariate effect whereas both Cox-MDR and AFT-MDR can adjust for confounding covariates in the frame of the regression model.

However, all these methods require a cross-validation procedure to identify the best SNP pairs among all possible combinations of SNPs and computationally intensive permutation testing to check the statistical significance for the identified SNP pairs as performed in the original MDR method. To overcome this shortcoming of MDR, the unified model-based multifactor dimensionality reduction (UM-MDR) method was proposed by unifying significance testing with the MDR algorithm in the frame of the regression model [10]. In the UM-MDR method, multi-level genotypes are classified into high- and low-risk groups, and an indicator variable for the high-risk group is defined in the first step. Then, significance testing is unified in the frame of the regression model by including this indicator variable for the high-risk group as one of covariates with other adjusting covariates. One of the advantages of UM-MDR is that it allows different types of classification rules in the first step. Thus, a simple approach, called the Cox-UMMDR [11], was recently proposed by plugging the Cox-MDR into the first step and combining the classified indicator with the significance testing procedure of the UM-MDR.

In this study, we propose two different simple methods, called KM-UMMDR and Cox2-UMMDR. The KM-UMMDR method uses the KM-MDR algorithm in the first step and combines it with the second step of UM-MDR. The Cox2-UMMDR modifies the classification step of Cox-UMMDR by allowing the covariate effect to be adjusted in the first step and fitting only one indicator variable for the high-risk group in the second step whereas the adjusting covariates are considered in the regression model in the second step for Cox-UMMDR. Throughout the simulation study, the powers of the five methods— KM-UMMDR, Cox2-UMMDR, Cox-UMMDR, Cox-MDR and KM-MDR— were compared under the two different scenarios mainly focusing on two-way interactions. One scenario compares the power of semi-parametric methods such as Cox- UMMDR, Cox2-UMMDR and Cox-MDR against that of nonparametric methods such as KM-UMMDR and KM-MDR by considering cases with and without a covariate effect. The other scenario compares the power of KM-UMMDR, Cox-UMMDR and Cox2-UMMDR against that of KM-MDR and Cox-MDR by considering cases with and without the main effect of SNPs. In addition, one simulation result is given under a three-way interaction model. Finally, both KM-UMMDR and Cox2-UMMDR were applied to a real dataset of Korean leukemia patients and a short discussion is provided.

## Methods

As described in Cox-UMMDR, there are two-step procedures for KM-UMMDR and Cox2-UMMDR. In the first step of KM-UMMDR, we classify the multi-genotypes into high- or low-risk groups using the Kaplan-Meier median survival time as in KM-MDR. For the two-way interaction model, all individuals are divided into nine groups with the same genotypes. We then compare the median survival time of each cell with the overall median survival time. If the median survival time of each cell is less than the overall median survival time, then the corresponding cell is classified as high-risk group. Otherwise, it is classified as low-risk group. Once all cells are classified as high(H) or low(L) risk groups, an H/L binary indicator, ***S***, is defined. In contrast, in the first step of Cox2-UMMDR, we classify the multi-genotypes into high- or low-risk groups using the sign of the sum of martingale residuals within each cell, where the martingale residuals are obtained from a reduced Cox model with covariates such as age, sex and other confounding variables. Cox2-UMMDR differs from Cox-UMMDR in the sense that the covariate effects are adjusted for in step 1 and then only an indicator variable, ***S***, is fitted in the regression model of step 2. By contrast, Cox-UMMDR does not adjust for the covariate effects in step 1 and fits a Cox model with all adjusting covariates and an H/L indicator ***S*** in step 2.

Once an indicator, ***S***, for the high-risk group is defined from the classification procedure in step 1, a similar procedure is implemented in step 2 for both KM-UMMDR and Cox2-UMMDR. In step 2, we fit the following two Cox models

$$ \lambda \left(t|S,Z\right)={\lambda}_0(t)\mathit{\exp}\left(\alpha S+\gamma Z\right) $$

for KM-UMMDR and

$$ \lambda \left(t|S\right)={\lambda}_0(t)\mathit{\exp}\left(\alpha S\right) $$

for Cox2-UMMDR.

Here, *λ*_0_(*t*) is a baseline hazard function, *S* is an indicator variable for the high-risk group, *Z* is the vector coding for the adjusting covariates, and α and *γ* are the corresponding parameters to *S* and *Z*, respectively. Because the median survival time is used as a classifier, the covariate effect cannot be adjusted for in step 1 of KM-UMMDR. However, KM-UMMDR adjusted for the covariate effect in step 2 by fitting the Cox model shown above, whereas KM-MDR cannot adjust for the covariate effect. In contrast, Cox2-UMMDR adjusted for the covariate effect in step 1 and fit a Cox model with only an indicator for the high-risk group shown above.

We tested the following null hypothesis: *H*_0_ : *α* = 0, i.e., whether the corresponding multi-locus is associated with the survival phenotype after adjusting for the covariate effect. To test the significance of the multi-locus model, a Wald type test statistic, $W={\hat{\alpha}}^2/\hat{Var}\left(\hat{\alpha}\right)$, was used, however, its asymptotic distribution followed a non-central chi-square distribution with one degree of freedom and the non-centrality parameter, *q*, as described in [10]. This is because *S* is defined as an indicator for the high-risk group after classification is performed in step 1. Because the mean of the non-central chi-square distribution is *q* + 1, we first estimate the mean, $\hat{\mu}$, of *W* under the null distribution and take $\hat{q}=\max \left(0,\hat{\mu}-1\right)$. As described in [10], we permute the trait a few times, for example, 5 or 10 times, and take the sample mean of the statistic *W* as $\hat{\mu}$. Here we can estimate the non-centrality parameter for each multi-locus model, or we can pool all the statistics and then estimate the common non-centrality parameter for all multi-locus models.

### Simulation study

Through simulation studies, we compared the power of the five methods— KM-UMMDR, Cox2-UMMDR, Cox-UMMDR, Cox-MDR and KM-MDR—under the two different scenarios. Scenario I compared the power of nonparametric methods with that of semi-parametric methods with and without adjusting for the covariate effect. As described above, KM-UMMDR and KM-MDR are nonparametric methods in the sense that the classification procedure is performed by the Kaplan-Meier median survival time in step 1, whereas Cox-MDR, Cox-UMDMR and Cox2-UMMDR are semi-parametric methods because they classify the martingale residuals from a Cox model with adjustment for the covariate effect. However, KM-UMMDR can adjust for covariate effects in the regression model in step 2 and can be regarded as a more improved approach than KM-MDR. Scenario II compares the power of original MDR methods, such as Cox-MDR and KM-MDR, with that of the unified model-based MDR methods, such as KM-UMMDR, Cox2-UMMDR and Cox-UMMDR without and with adjustment for marginal SNP effects.

First, we focus on a two-way interaction model and consider two disease-causal SNPs among 10 unlinked diallelic loci with the assumption of Hardy-Weinberg equilibrium and linkage equilibrium. For covariate adjustment, we consider only one covariate associated with survival time but with no interactions with any SNPs. We generated simulation datasets from different penetrance functions [12], which define a probabilistic relationship between the high- or low-risk status and SNPs. We then considered 14 different combinations of two different minor allele frequencies of (0.2, 0.4) and seven different heritability values of (0.01, 0.025, 0.05, 0.1, 0.2, 0.3, and 0.4). For each of the 14 heritability and minor allele frequency combinations, a total of five models were generated, yielding 70 epistasis models with various penetrance functions, as described in [12].

Let *f*_*ij*_ be an element from the *i*^*th*^ row and the *j*^*th*^ column of a penetrance function. Assuming that SNP1 and SNP2 are two disease-causal SNPs, we obtain the following penetrance function:

$$ {\boldsymbol{f}}_{\boldsymbol{ij}}=\boldsymbol{P}\left(\boldsymbol{high}\ \boldsymbol{risk}\ |\boldsymbol{SNP}\mathbf{1}=\boldsymbol{i},\boldsymbol{SNP}\mathbf{2}=\boldsymbol{j}\right) $$

We generated data of 400 patients from each of the 70 penetrance models to create one simulated dataset and repeated this procedure 100 times. We generated the survival time from a Cox model, which can be specified as follows:

$$ \boldsymbol{\lambda} \left(\boldsymbol{t}|\boldsymbol{x},\boldsymbol{z}\right)={\boldsymbol{\lambda}}_{\mathbf{0}}\left(\boldsymbol{t}\right)\boldsymbol{\exp}\left(\boldsymbol{\alpha} \boldsymbol{x}+\boldsymbol{\gamma} \boldsymbol{z}\right) $$

Here ***x*** is an indicator variable with value 1 for a high-risk group and 0 for a low-risk group. We set ***α =*** **0.8*, γ =*** **0.8** and *z* as the adjusting covariate generated from ***N***(**0**, **1**). In addition, the baseline hazard function follows a Weibull distribution with a shape parameter of 5 and a scale parameter of 2, the censoring time being generated from a uniform distribution, *U*(0,*c*) depending on the censoring fraction (0.1, 0.3).

First, we calculated the Type I error to check the validity of the Cox2-UMMDR and KM-UMMDR methods for which data were generated under the null hypothesis where *H*_0_ : ***α*** = 0, across various heritability and minor allele frequencies (MAF). We generated 1000 null dataset with eight non-causal SNPs and the Type I error of the selection rate of each SNP pair under the null model became $\frac{1}{\left(\genfrac{}{}{0pt}{}{8}{2}\right)}=0.0357$. We considered five different MAFs as 0.05, 0.1, 0.2, 0.3, and 0.4 and four different censoring fractions (CF) as 0.0, 0.1, 0.3, and 0.5. The selected values of MAF are appropriate in the simulation study for GWAS because the alleles with MAF less than 0.05 is regarded as rare variant. For example, in the studies on sudden cardiac arrest and systemic lupus erythematosus, the number of SNPs have low MAFs ( < 0.1 or 0.1≤ MAFs <0.2) [13]  In contrast, many age-at-onset of SNPs have high MAF (0.2< MAF <0.4), which are significantly associated with late onset Alzheimer disease [14]. Table 1 shows the Type I error of the Cox2-UMMDR and KM-UMMDR methods across various combinations of MAF and CF. Although most cases showed a conservative trend, it is concluded that the Type I error is well controlled with being less than 0.035.

**Table 1:** Type I error of 2-way Cox2-UMMDR and KM-UMMDR for the combinations of MAF and CF.

MAF	Cox2-UMMDR				KM-UMMDR			
	CF = 0.0	CF = 0.1	CF = 0.3	CF = 0.5	CF = 0.0	CF = 0.1	CF = 0.3	CF = 0.5
0.05	0.025	0.021	0.022	0.019	0.029	0.021	0.026	0.024
0.1	0.023	0.024	0.022	0.021	0.027	0.022	0.024	0.027
0.2	0.024	0.022	0.023	0.029	0.023	0.023	0.027	0.029
0.3	0.024	0.023	0.028	0.027	0.023	0.027	0.028	0.028
0.4	0.026	0.023	0.027	0.021	0.024	0.026	0.024	0.031

For the power comparison, we consider two different scenarios for the simulation study. In Scenario I, the survival times are generated from the Cox model as follows:

$$ \boldsymbol{\lambda} \left(\boldsymbol{t}|\boldsymbol{x},\boldsymbol{z}\right)={\boldsymbol{\lambda}}_{\mathbf{0}}\left(\boldsymbol{t}\right)\mathbf{\exp}\left(\boldsymbol{\alpha} \boldsymbol{x}+\boldsymbol{\gamma} \boldsymbol{z}\right) $$

where ***α*** = **0.8**, ***γ*** = **0.0** and ***α*** = **0.8**, ***γ*** = **0.8**, respectively.

In Scenario II, the survival times were generated from the Cox model as follows:

$$ \boldsymbol{\lambda} \left(\boldsymbol{t}|\boldsymbol{x},\boldsymbol{z},\boldsymbol{SNP}\mathbf{3}\right)={\boldsymbol{\lambda}}_{\mathbf{0}}\left(\boldsymbol{t}\right)\mathbf{\exp}\left(\boldsymbol{\alpha} \boldsymbol{x}+\boldsymbol{\gamma} \boldsymbol{z}+\boldsymbol{\delta} \boldsymbol{SNP}\mathbf{3}\right) $$

where ***α =*** **0.8*, γ =*** **0.8*, δ =*** **0.5** and ***α =*** **0.8*, γ =*** **0.0*, δ =*** **0.5**. Here ***δ*** denotes the marginal main effect of *SNP3* on the hazard rate. In addition, we consider a three-way interaction model to compare the power of the five methods, similar to the two-way interaction model. For the three-way interaction model, we use penetrance functions, ***P***(***high risk***|***AAbbcc***) ***=*** **0.2*, P***(***high risk***| ***AaBbcc***) ***=*** **0.2*, P***(***high risk***| ***aaBBcc***) ***=*** **0.2*, P***(***high risk***| ***aaBbCc***) ***=*** **0.2*, P***(***high risk***| ***AabbCc***) ***=*** **0.2*, P***(***high risk***| ***aabbCC***) ***=*** **0.2** as suggested by Ritchie et al. [5]. Here, we assume that SNP1, SNP2 and SNP3 are the three disease-causal SNPs and each has diallelic locus of (***A***, ***a***)***,*** (***B***, ***b***)***,*** (***C***, ***c***). Similar to the two-way interaction models, we generated data of 400 patients from the penetrance model to create one simulated dataset and repeated this procedure 100 times. The survival time was generated from a Cox model specified as follows:

$$ \boldsymbol{\lambda} \left(\boldsymbol{t}|\boldsymbol{x},\boldsymbol{z},\boldsymbol{SNP}\mathbf{4}\right)={\boldsymbol{\lambda}}_{\mathbf{0}}\left(\boldsymbol{t}\right)\mathbf{\exp}\left(\boldsymbol{\alpha} \boldsymbol{x}+\boldsymbol{\gamma} \boldsymbol{z}+\boldsymbol{\delta} \boldsymbol{SNP}\mathbf{4}\right) $$

Here, ***α*** was set to be 1.2, and ***γ*** and ***δ*** had four different combinations (i) [***γ***, ***δ***] = [1.2, 0.5], (ii) [***γ***, ***δ***] = [1.2, 0.0], (iii) [***γ***, ***δ***] = [0.0,0.5] and (iv) [***γ***, ***δ***] = [0.0, 0.0].

**Fig. 1:**
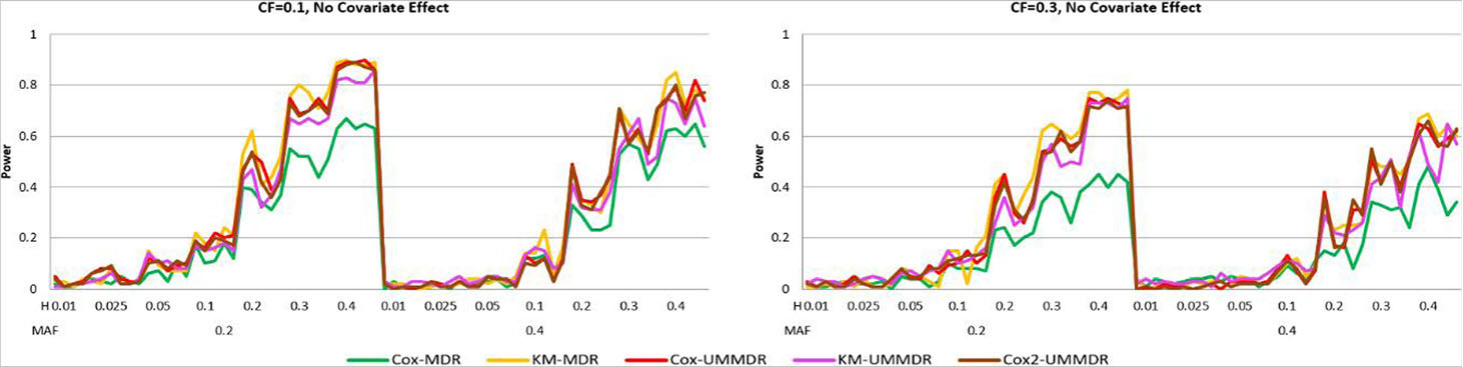
Power curves of five methods under α = 0.8, γ = 0.0

First, we consider the simulation result for the two-way interaction model under Scenario I. Figure 1 shows the power curve of the five methods under *α* = 0.8, *γ* = 0.0 where there is no covariate effect and the CFs are 0.1 and 0.3. The x-axis represents 70 models by ordering the combination of MAF and heritability, where 5 different models are given for each combination of MAF = (0.2, 0.4) and H = (0.01, 0.025, 0.05, 0.1, 0.2, 0.3, 0.4). whereas the y-axis represents the power of the five methods across 70 different penetrance models [12].

As shown in Fig. 1, the power decreases as the CF increases whereas the power is greater when MAF = 0.2 than when MAF = 0.4. Under the no covariate effect model, KM-UMMDR, KM-MDR, Cox-UMMDR, and Cox2-UMMDR performed similarly, and their powers increased as heritability increased, except for Cox-MDR. It is expected that nonparametric approaches, such as KM-UMMDR and KM-MDR, are more powerful than semi-parametric approaches, such as Cox-UMMDR, Cox2-UMMDR, and Cox-MDR. However, Cox-UMMDR and Cox2-UMMDR seem to be very close to KM-UMMDR and KM-MDR in terms of the power curve.

**Fig. 2:**
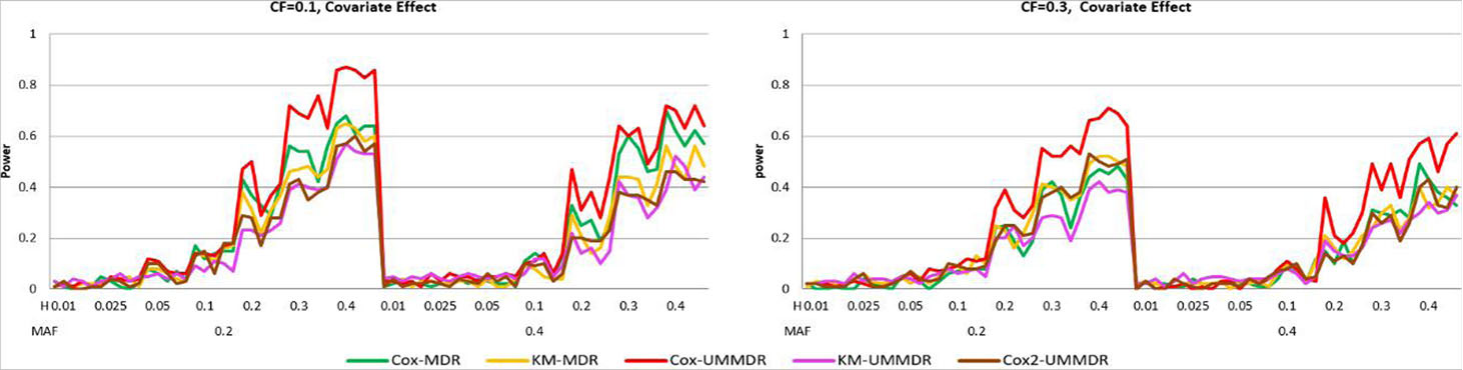
Power curves of five methods under α = 0.8, γ = 0.8

Figure 2 shows the power curve of the five methods under ***α =*** **0.8*, γ =*** **0.8*,*** where there is a confounding covariate effect. As shown in Fig. 2, Cox-UMMDR has the highest power, however, the performance of Cox-MDR is similar to that of KM-UMMDR, KM-MDR, and Cox2-UMMDR, except when MAF = 0.4, and the CF is 0.1. It is interesting that Cox2-UMMDR behaves almost the same as KM-UMMDR, although Cox2-UMMDR adjusted for the covariate effects in step 1. This implies that the effect of covariates should be adjusted for in step 2 rather than in step 1 because Cox-UMMDR is more powerful than Cox2-UMMDR. The only difference between Cox-UMMDR and Cox2-UMMDR is when the effects of covariates are considered.

**Fig. 3:**
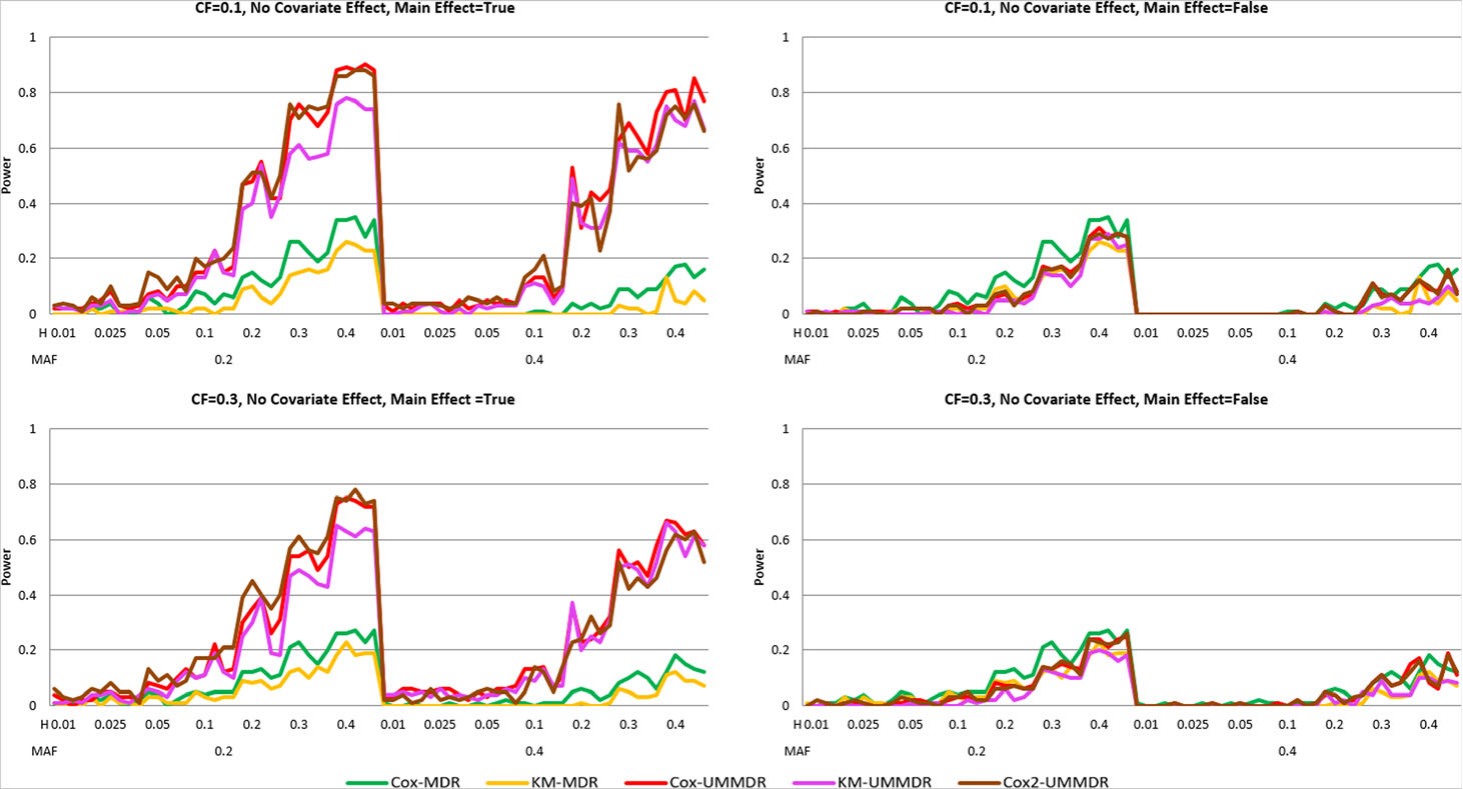
Power curves of five methods under α = 0.8, γ = 0.0, δ = 0.5

Second, we consider the simulation result for the two-way interaction model under Scenario II. Because a non-causal SNP effect exists in a true model, we compare two different cases where the main effect of SNPs is adjusted for (main = True) and not adjusted for (main = False). Figure 3 shows the power curve of the five methods under ***α =*** **0.8*, γ =*** **0.0*, δ =*** **0.5**, where there is no covariate effect, but there exists a non-causal SNP main effect. As shown in Fig. 3, the powers of all methods were very low when the main effect of SNPs is not adjusted for regardless of any combination of MAF, heritability, and CF. However, when the main effect of SNPs is adjusted for, the powers of Cox-UMMDR, Cox2-UMMDR and KM-UMMDR are relatively higher than those of Cox-MDR and KM-MDR. This implies that adjusting for the main effects of causal SNPs helps to identify the interaction effect of these SNPs although there exists a non-causal SNP main effect. In summary, three methods based on UM-MDR are more powerful than two methods based on the original MDR method when there exists an SNP main effect and it is properly adjusted for.

**Fig. 4:**
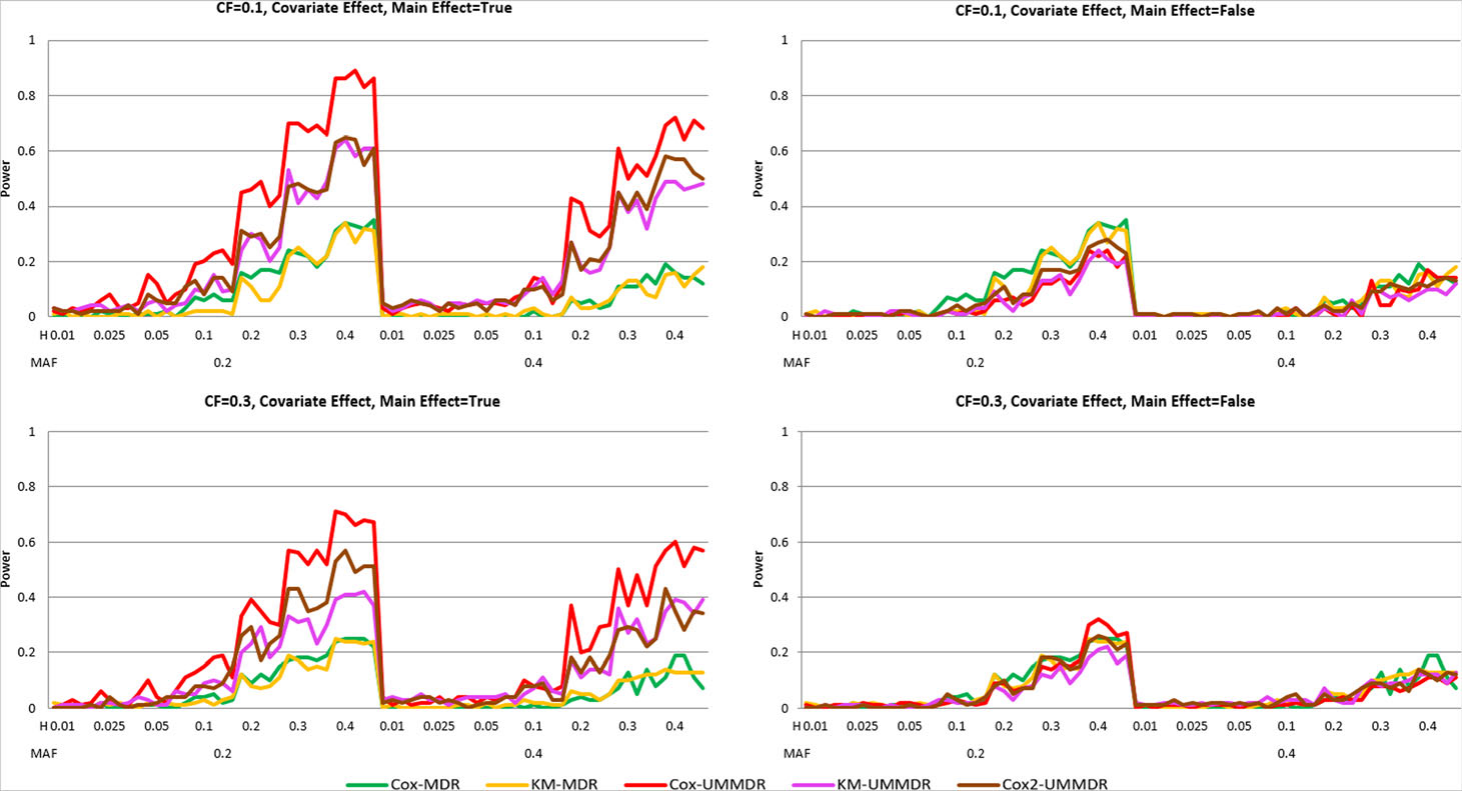
Power curves of five methods under α = 0.8, γ = 0.8, δ = 0.5

Figure 4 shows the power curve of the five methods under ***α =*** **0.8*, γ =*** **0.8*, δ =*** **0.5**, where there exists a non-causal SNP main effect as well as a confounding covariate effect. As shown in Fig. 3, the power of all methods is very low when the main effect of an SNP is not adjusted for (main = False). However, when the main effect of an SNP is adjusted for (main = True), the power of Cox-UMMDR is substantially greater than that of Cox2-UMMDR and KM-UMMDR. As mentioned previously, it is more efficient that the effect of confounding covariates should be adjusted for in step 2 than in step 1. Both Cox2-UMMDR and KM-UMMDR were very similar when the CF was 0.1, whereas Cox2-UMMDR had a relatively greater power than KM-UMMDR when the CF was 0.3. Similar to Fig. 3, the powers of Cox-MDR and KM-MDR were noticeably lower than those of Cox-UMMDR, Cox2-UMMDR, and KM-UMMDR.

Finally, we consider a three-way interaction model that has only one type of penetrance function: *P*(*high risk*|*AAbbcc*) = 0.2, *P*(*high risk*| *AaBbcc*) = 0.2, *P*(*high risk*| *aaBBcc*) = 0.2, *P*(*high risk*| *aaBbCc*) = 0.2, *P*(*high risk*| *AabbCc*) = 0.2,

*P*(*high risk*| *aabbCC*) = 0.2, as suggested by Ritchie et al. [5].

We calculated the Type I error to check the validity of the Cox2-UMMDR and KM-UMMDR methods for which data were generated under the null hypothesis where *H*_0_ : ***α*** = 0, across various heritability and minor allele frequencies (MAF). We generated 1000 null dataset with six non-causal SNPs and the Type I error of the selection rate of each SNP pair under the null model became $\frac{1}{\left(\genfrac{}{}{0pt}{}{6}{2}\right)}=0.05$. Table 2 shows the Type I errors of the Cox2-UMMDR and KM-UMMDR methods across various combinations of MAF and CF. Although most cases showed a conservative trend, it is concluded that the Type I error is well controlled.

**Table 2:** Type I error of 3-way Cox2-UMMDR and KM-UMMDR for the combinations of MAF and CF.

MAF	Cox2-UMMDR				KM-UMMDR			
	CF = 0.0	CF = 0.1	CF = 0.3	CF = 0.5	CF = 0.0	CF = 0.1	CF = 0.3	CF = 0.5
0.05	0.045	0.041	0.032	0.038	0.039	0.033	0.033	0.037
0.1	0.043	0.044	0.042	0.040	0.037	0.034	0.034	0.034
0.2	0.044	0.032	0.033	0.039	0.043	0.033	0.039	0.035
0.3	0.031	0.033	0.038	0.046	0.043	0.034	0.037	0.039
0.4	0.036	0.033	0.037	0.043	0.044	0.036	0.034	0.039

**Fig. 5:**
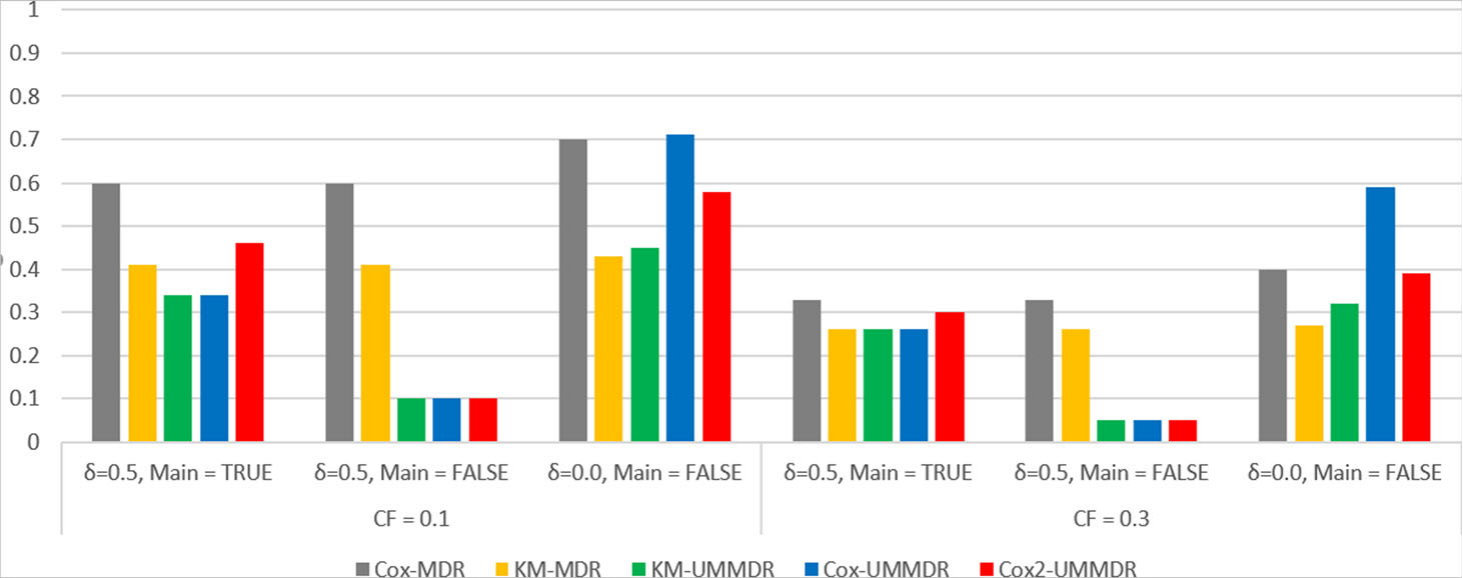
Power curves of the five methods under **γ = 1.2**

**Fig. 6:**
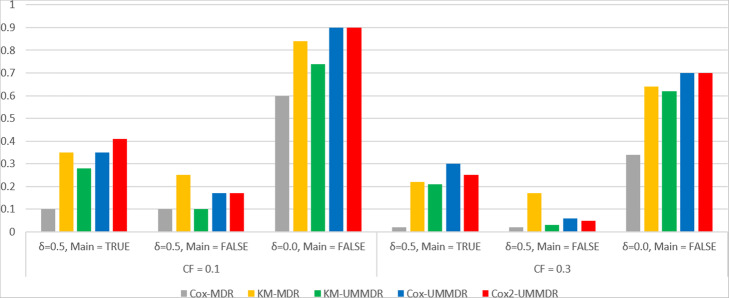
Power curves of the five methods under **γ = 0.0**

For the power comparison, we considered four different combinations of the covariate effect and SNP main effect: γ (1.2 and 0.0) and δ (0.5 and 0.0), similar to the simulation study for the two-way interaction. Figures 5 and 6 show the bar plots for power comparison of the five methods in both cases with and without covariate effect. When there exists a covariate effect (that is, γ = 1.2), Cox-MDR, Cox-UMMDR and Cox2-UMMDR have relatively greater powers than KM-MDR and KM-UMMDR. When there is an SNP main effect (that is, δ = 0.5), the powers of KM-UMMDR, Cox-UMMDR, and Cox2-UMMDR were relatively greater when it is properly adjusted for than when it is not adjusted for. Interestingly, the powers of Cox-MDR and KM-MDR were higher than those of KM-UMMDR, Cox-UMMDR, and Cox2-UMMDR when both the covariate effect and SNP main effect exist (that is, γ = 1.2, δ = 0.5). This implies that the main effect of the SNP may be reflected as any covariate effect. However, the power of Cox-MDR hardly decreases when only the main effect of SNP (that is, γ = 0.0, δ = 0.5) is considered and is properly adjusted for. Note that the powers of KM-MDR, Cox-UMMDR, and Cox2-UMMDR were similar higher to that of Cox-MDR when there was neither a covariate effect nor the SNP main effect (that is, γ = δ = 0.0).

### Real data analysis

We analyzed a real dataset consisting of 97 Korean Acute Myeloid Leukemia (AML) patients with demographic information of age and sex, and genetic information of 139 SNPs. At the end of the study, 40 deaths occurred, and 57 patients were alive. We applied the two proposed methods of KM-UMMDR and Cox2-UMMDR to detect SNP-SNP interactions associated with survival time by adjusting for age and sex. As pointed by a referee, we tested the proportional hazards assumption for a Cox model with two covariates of age and sex [15]. The chi-squared test statistic has a value of 4.69 with degrees of freedom of 2, which corresponds to the *p*-value of 0.095. It can be concluded that the proportional hazards assumption is satisfied.

We fitted a univariate Cox model with each SNP by adjusting for age and sex to consider the marginal effect of SNP. We found that 21 SNPs have significant marginal effects on survival time. To summarize these marginal effects of 21 SNPs, we performed the principle component analysis and took two principal components (PC) as a covariate, which accounted for 78% of the variation. We considered four different models in identifying gene-gene interactions by KM-UMMDR and Cox2-UMMDR as follows:

(1) PC unadjusted and main effects of SNP1 and SNP2 unadjusted:

$$ \boldsymbol{\lambda} \left(\boldsymbol{t}|\boldsymbol{S},\boldsymbol{age},\boldsymbol{sex}\right)={\boldsymbol{\lambda}}_{\mathbf{0}}\left(\boldsymbol{t}\right)\boldsymbol{\exp}\left(\boldsymbol{\beta} \boldsymbol{S}+{\boldsymbol{\gamma}}_{\mathbf{1}}\boldsymbol{age}+{\boldsymbol{\gamma}}_{\mathbf{2}}\boldsymbol{sex}\right) $$

(2) PC adjusted and main effects of SNP1 and SNP2 unadjusted:

$$ \boldsymbol{\lambda} \left(\boldsymbol{t}|\boldsymbol{S},\boldsymbol{age},\boldsymbol{sex},{\boldsymbol{PC}}_{\mathbf{1}},{\boldsymbol{PC}}_{\mathbf{2}}\right)={\boldsymbol{\lambda}}_{\mathbf{0}}\left(\boldsymbol{t}\right)\mathbf{\exp}\left(\boldsymbol{\beta} \boldsymbol{S}+{\boldsymbol{\gamma}}_{\mathbf{1}}\boldsymbol{age}+{\boldsymbol{\gamma}}_{\mathbf{2}}\boldsymbol{sex}+{\boldsymbol{\delta}}_{\mathbf{1}}\boldsymbol{P}{\boldsymbol{C}}_{\mathbf{1}}+{\boldsymbol{\delta}}_{\mathbf{2}}\boldsymbol{P}{\boldsymbol{C}}_{\mathbf{2}}\right) $$

(3) PC unadjusted and main effects of SNP1 and SNP2 adjusted:

$$ \boldsymbol{\lambda} \left(\boldsymbol{t}|\boldsymbol{S},\boldsymbol{age},\boldsymbol{sex},\boldsymbol{SNP}\mathbf{1},\boldsymbol{SNP}\mathbf{2}\right)={\boldsymbol{\lambda}}_{\mathbf{0}}\left(\boldsymbol{t}\right)\mathbf{\exp}\left(\boldsymbol{\beta} \boldsymbol{S}+{\boldsymbol{\gamma}}_{\mathbf{1}}\boldsymbol{age}+{\boldsymbol{\gamma}}_{\mathbf{2}}\boldsymbol{sex}+{\boldsymbol{\theta}}_{\mathbf{1}}\boldsymbol{SNP}\mathbf{1}+{\boldsymbol{\theta}}_{\mathbf{2}}\boldsymbol{SNP}\mathbf{2}\right) $$

(4) PC adjusted and main effects of SNP1 and SNP2 adjusted:

$$ \boldsymbol{\lambda} \left(\boldsymbol{t}|\boldsymbol{S},\boldsymbol{age},\boldsymbol{sex},{\boldsymbol{PC}}_{\mathbf{1}},{\boldsymbol{PC}}_{\mathbf{2}},\boldsymbol{SNP}\mathbf{1},\boldsymbol{SNP}\mathbf{2}\right)={\boldsymbol{\lambda}}_{\mathbf{0}}\left(\boldsymbol{t}\right)\mathbf{\exp}\left(\boldsymbol{\beta} \boldsymbol{S}+{\boldsymbol{\gamma}}_{\mathbf{1}}\boldsymbol{age}+{\boldsymbol{\gamma}}_{\mathbf{2}}\boldsymbol{sex}+{\boldsymbol{\delta}}_{\mathbf{1}}\boldsymbol{P}{\boldsymbol{C}}_{\mathbf{1}}+{\boldsymbol{\delta}}_{\mathbf{2}}\boldsymbol{P}{\boldsymbol{C}}_{\mathbf{2}}+{\boldsymbol{\theta}}_{\mathbf{1}}\boldsymbol{SNP}\mathbf{1}+{\boldsymbol{\theta}}_{\mathbf{2}}\boldsymbol{SNP}\mathbf{2}\right) $$

**Fig. 7:**
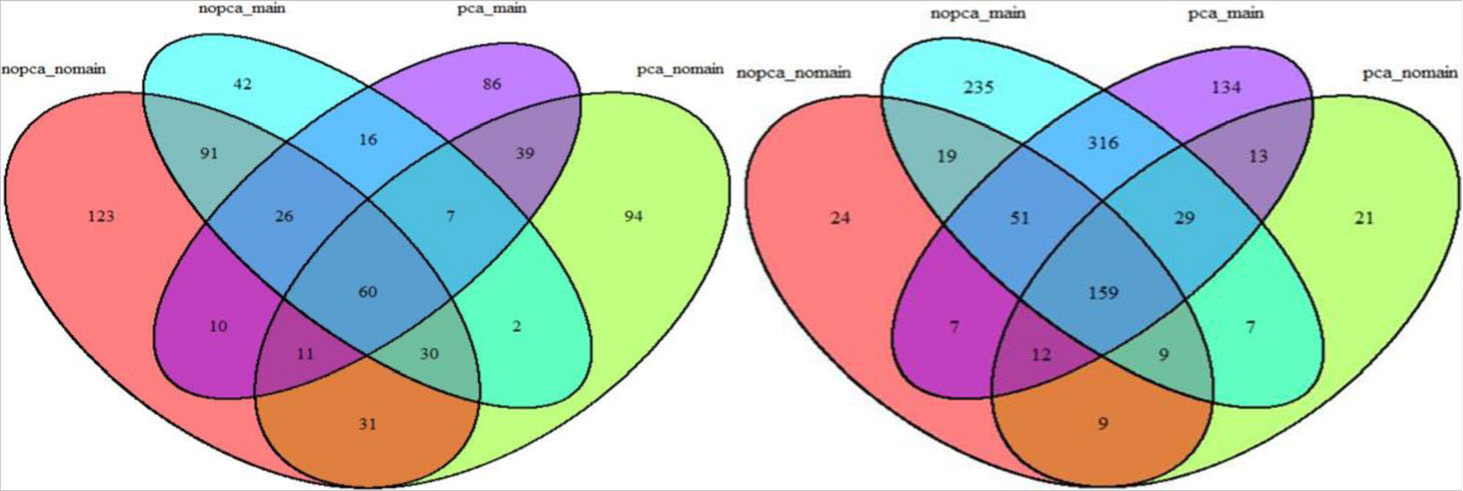
Venn Diagram for the number of significant 2-way SNP pairs identified by Cox2-UMMDR(left) and KM-UMMDR(right)

We obtained a list of significant SNP pairs from each model, and summarized their numbers using a Venn diagram. Figure 7 shows the Venn Diagrams for the number of significant two-way SNP pairs identified by Cox2-UMMDR (left) and KM-UMMDR (right). The number of significant SNP pairs differed by these two methods under the four different models. We show that 159 significant SNP pairs were overlapped by four different models under KM-UMMDR whereas only 60 significant SNP pairs were overlapped by four different models under Cox2-UMMDR. In addition, although the number of SNP pairs varied across the four models, 40 SNP pairs were found to be commonly significant by both Cox2-UMMDR and KM-UMMDR.

Among these SNP pairs, we chose the best two SNP pairs that yielded the H/L indicator and checked their *p*-value from the fitted Cox model, adjusting for age and sex in step 2 as shown in Table 3. The SNP pair (“**rs580032”, “rs1960207”)** were present in the list of the best SNP pairs obtained by both Cox2-UMMDR and KM-UMMDR, and a p-value less than 0.001 was obtained.

**Table 3:** Significance test for the interaction effects of top two SNP pairs identified by Cox2-UMMDR and KM-UMMDR

Method	SNP1 SNP2	***P***-value
**KM UM-MDR**	**rs1960207 rs2917669** **rs580032 rs1960207**	**0.0000003** **0.0002346**
**Cox2 UM-MDR**	**rs580032 rs1960207** **rs17170153 rs1801133**	**0.0001942** **0.0000826**

**Fig. 8:**
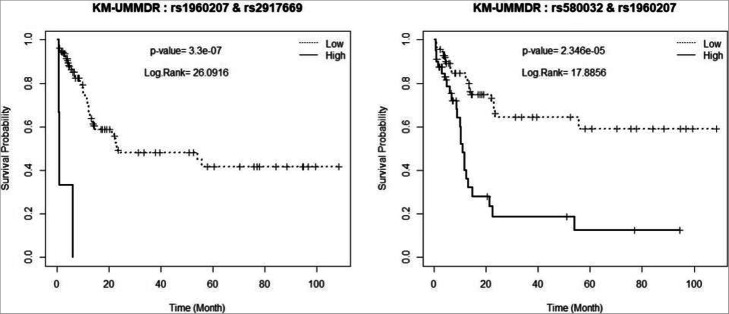
Kaplan-Meier survival plots attributed by SNP pairs from KM-UMMDR

**Fig. 9:**
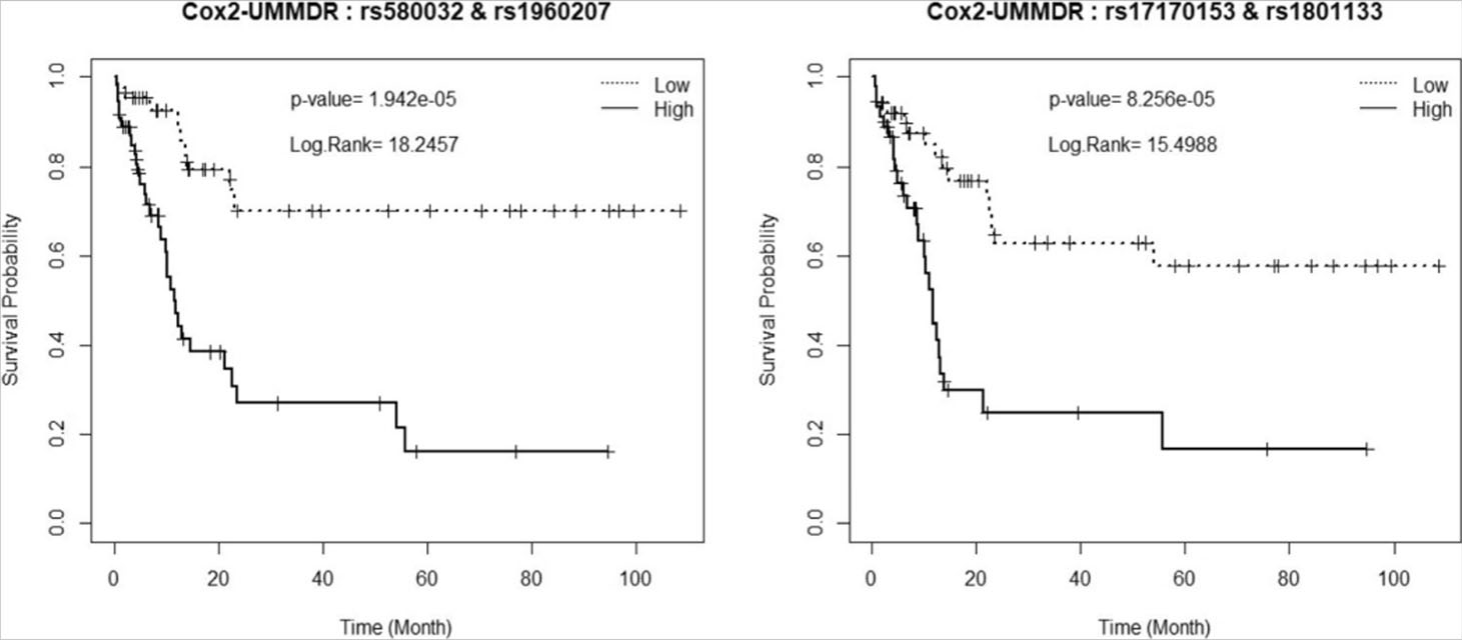
Kaplan-Meier survival plots attributed by SNP pairs from Cox2-UMMDR

In addition, we provide the Kaplan-Meier survival plots of these two groups that were divided with respect to the H/L indicator attributed to the corresponding SNP pairs, as shown in Figs. 8 and 9. In other words, both ‘high-risk’ and ‘low-risk’ groups were defined via step 1 classification using the best SNP pairs obtained by KM-MDR and the modified Cox-MDR, respectively. It is shown that the identified SNP pairs could separate the two survival plots significantly, which implies that there may be two-way interaction effects associated with the survival phenotype.

As shown in Fig. 8, the SNP pair (‘rs196020’, ‘rs2917669’) was present in the extremely sparse high-risk group, which consists of only 3 individuals and the others belong to the low-risk group. This would be due to sparsity of a specific genotype in a small sample. The genotypes of these three individuals who belong to the high-risk group are DCTD ‘rs1960207’ (GA genotype) in combination with NQO1 ‘rs2917669’ (TT genotype). Among these two SNPs, NQO1 ‘rs2917669’ was listed in the genetic polymorphism as risk factors of early-onset lung cancer [16]. Even though there is a large imbalance between the high- and low-risk groups, the survival curve for the high-risk group was significantly lower than that for the low-risk group. The SNP pair (“rs580032”, “rs1960207”) was selected as one of the best top two pairs by both KM-UMMDR and Cox2-UMMDR. However, the two KM-plots were not the same and the corresponding log-rank test statistics were also different at 17.8856 and 18.2457, respectively. This implies that KM-UMMDR and Cox2-UMMDR classify the high- and low-risk groups differently in step 1 because the former uses the median survival time and the latter uses the martingale residual, respectively. In fact, one cell among all combinations of two SNPs (“rs580032”, “rs1960207”) is classified as high-risk by KM-UMMDR but as low-risk by Cox2-UMMDR. However, the corresponding cell have only three individuals and provides very similar values to the log-rank test statistics.

## Discussion

We proposed two simple methods, KM-UMMDR and Cox2-UMMDR, by adopting the classification rule from KM-MDR and the modified Cox-MDR. These methods are extensions of KM-MDR and Cox-MDR to UM-MDR, similar to Cox-UMMDR. In this study, we focused on the comparison of the power of the five different methods such as KM-UMMDR, Cox2-UMMDR, Cox-UMMDR, Cox-MDR, and KM-MDR across various scenarios.

Among those, both Cox-MDR and KM-MDR need cross-validation and permutation testing to identify significant interaction models whereas Cox-UMMDR, KM-UMMDR, and Cox2-UMMDR provide the significance of the interaction model in the frame of the regression model without any intensive computing. In contrast, both KM-MDR and KM-UMMDR are nonparametric approaches in which any covariate effect cannot be adjusted for in the classification procedure, whereas Cox-MDR, Cox-UMMDR, and Cox2-UMMDR can adjust for the covariate effect. However, the covariate effect is considered in the procedure of testing for the high-risk group classified by KM-UMMDR in the regression model in step 2. Therefore, KM-UMMDR is more flexible than KM-MDR in the sense that the former can adjust for the covariate effect and is expected to be associated with reduced computing time.

Comparing Cox2-UMMDR with Cox-UMMDR, the simulation results show that Cox-UMMDR is more powerful than Cox2-UMMDR, which implies that the effect of covariates should be adjusted for in the regression model rather than in the classification procedure. Furthermore, when there exists any SNP main effect, it should be properly adjusted for in the regression model because the powers of Cox-UMMDR, KM-UMMDR, and Cox2-UMMDR are very low when it is not adjusted for. Throughout the simulation results, KM-UMMDR, Cox2-UMMDR, and Cox-UMMDR were more powerful than Cox-MDR and KM-MDR when there were any main effects of SNPs.

We performed a simulation study for only one three-way interaction model because few higher-order interaction models are available for simulation studies. Although only one model is provided in the simulation study, the power trend seems to be similar to that of the two-way interaction model. Focusing on the two-way interaction model, Cox-UMMDR is the most powerful, except for a few cases among the five methods whereas KM-UMMDR and Cox2-UMMDR have reasonable power in most cases.

As pointed out by a referee, we should focus on large scale data because the proposed method is for identifying gene-gene interaction associated with the survival phenotype in GWAS. We tried to implement the additional simulations for the large dataset to investigate whether there is the same trend for GWAS as shown in the simulation results for small dataset. Due to intensive computing time, we conducted a simulation study under the limited setting for *n* = 800, p = 50, and CF = 0.1, 0.3, and γ = 0.0. In addition, we randomly chose 8 models among 70 models according to the combination of two of MAF (0.2 and 0.4) and four of heritability (0.1, 0.2, 0.3 and 0.4) for 2-way interaction model. Although the results are not given here, we found that the trend of the power for larger sample size and larger number of SNPs is similar to that shown in Fig. 1, which implies that the order of the magnitude of the five methods has not changed significantly.

As shown in the power comparison, the power of the five methods tends to reach to a recommended threshold, e.g., 80%, when the censoring fraction is low and heritability is 0.4. However, it is common to have higher censoring fraction than 0.1 in survival analysis and lower SNP-based heritability than 0.4 in complex diseases. To overcome this limitation, it could be possible to increase the sample size and reduce the heterogeneity among samples by adjusting for the confounding factors. In this simulation study, we set the effect of causal SNP is α = 0.8, but the power was shown to be larger if the effect size increases to α = 1.0 or α = 1.2 [9, 10].

In this study, we only consider a Cox regression model in step 2 but it is possible to fit any other regression model such as an AFT model when the proportional hazard assumption is not valid for some covariates. For example, multi-genotypes are classified into high- and low- risk groups by KM-MDR in step 1 and fit an AFT regression model with an indicator of the high-risk group and the other adjusting covariates in step 2. By testing the significance of the indicator for the high-risk group, we can show that SNPs have a high-order interaction effect on the survival phenotype.

## Data Availability

The real data of Korean leukemia patients is not available. The R-program for KM-UMMDR and Cox2-UMMDR is available at http://github.com/leesy80/Seungylee/coxumMDR
